# Combination Therapy of High-Dose Rabeprazole Plus Metronomic Capecitabine in Advanced Gastro-Intestinal Cancer: A Randomized Phase II Trial

**DOI:** 10.3390/cancers12113084

**Published:** 2020-10-22

**Authors:** Michela Roberto, Adriana Romiti, Federica Mazzuca, Annalisa Milano, Chiara D’Antonio, Luana Lionetto, Rosa Falcone, Lidia Strigari, Maurizio Simmaco, Stefano Fais, Paolo Marchetti

**Affiliations:** 1 Department of Clinical and Molecular Medicine, University “La Sapienza”, 00189 Roma, Italy; michela.roberto@uniroma1.it (M.R.); adriana.romiti@uniroma1.it (A.R.); paolo.marchetti@uniroma1.it (P.M.); 2 Oncology Unit, Sant’ Andrea University Hospital, 00189 Roma, Italy; annalisa.milano@ospedalesantandrea.it (A.M.); cdantonio@ospedalesantandrea.it (C.D.); 3 Mass Spectrometry Lab-Clinical Biochemistry Unit, Sant’Andrea University Hospital, 00189 Rome, Italy; llionetto@ospedalesantandrea.it; 4 Department of Translational and Precision medicine, University “La Sapienza”, viale dell’Università 37, 00185 Rome, Italy; rosa.falcone@uniroma1.it; 5 Department of Medical physics, Policlinico S.Orsola Malpighi, 40138 Bologna, Italy; lidia.strigari@aosp.bo.it; 6 Department of Neurosciences, Mental Health and Sensory Organs, University “La Sapienza”, via di Grottarossa 1035, 00189 Roma, Italy; maurizio.simmaco@uniroma1.it; 7 Unit of Laboratory and Advanced Molecular Diagnostic, Sant’Andrea University Hospital, 00189 Rome, Italy; 8 Department of Oncology and Molecular Medicine, National Institute of Health, Viale Regina Elena 299, 00161 Rome, Italy; stefano.fais@iss.it

**Keywords:** combination therapy, proton-pump inhibitors (PPIs), metronomic capecitabine, gastrointestinal cancer, drug repurposing

## Abstract

**Simple Summary:**

This is the first phase II study of high dose rabeprazole repurposing (1.5 mg/kg bid, three days a week) combined with metronomic capecitabine (mCAP), 1500 mg/daily, in gastrointestinal cancer, aimed at evaluating the activity and safety of high-dose proton pump inhibitor in combination with mCAP as salvage treatment in pretreated patients. A 3-months PFS rate of 66% and 57% was reported in the mCAP-rabeprazole and mCAP group, respectively. Although, the adjunct of high dose rabeprazole to mCAP did not improve mCAP activity, the combination of proton pump inhibitor with chemotherapy would deserve to be further investigated.

**Abstract:**

Background: In recent years, proton pump inhibitors (PPIs) have been investigated at high-dose to modulate tumor microenvironment acidification thus restoring chemotherapeutic sensitivity. This is the first trial to study activity and safety of repurposing high dose rabeprazole combined with metronomic capecitabine (mCAP). Methods: A phase II study in which patients with gastrointestinal cancer, refractory to standard treatments, who had a life expectancy >3 months, were blind randomized 1:1 to mCAP, 1500 mg/daily, continuously with or without rabeprazole 1.5 mg/kg bid, three days a week. The primary endpoint was 3-months progression-free survival (PFS). The secondary endpoints were clinical benefit (CB) and overall survival (OS). Safety and plasma concentrations of capecitabine and its metabolites (5′-DFUR and 5-FU) were also evaluated. Results: Sixty-seven (median age 69 years; 63% male; 84% colorectal cancer, 76% ECOG-PS ≤ 1; 84% pretreated with two or more lines of chemotherapy) out of 90 patients screened for eligibility, were randomized to receive mCAP+rabeprazole (*n* = 32) vs. mCAP (*n* = 35). All patients were evaluable for response. No significant difference between mCAP+rabeprazole vs. mCAP, in terms of 3-months PFS rate (HR = 1.43, 95%CI 0.53–3.85; *p* = 0.477), median PFS (HR = 1.22, 95%CI 0.75–2.00, *p* = 0.420), CB (RR = 0.85, 95%CI 0.29–2.44; *p* = 0.786) and median OS (HR = 0.89, 95%CI 0.54–1.48; *p* = 0.664) was observed. However, a 3-year OS rate of 10% and 12% was reported in the mCAP-rabeprazole and mCAP groups, respectively. Overall, no grade 3 or 4 toxicity occurred but grade 1 or 2 adverse event of any type were more frequently in the mCAP+rabeprazole group than in the mCAP (OR 2.83, 95%CI 1.03–7.79; *p* = 0.043). Finally, there was not statistically significant difference in the plasma concentration of capecitabine and its metabolites between the two groups. Conclusions: Although the adjunct of high dose rabeprazole to mCAP was not shown to affect mCAP activity, as PPI are being investigated worldwide as drugs to be repositioned in cancer treatment and also considering the limited sample size as well as the favorable safety profile of the combination in the present study, further clinical investigations are desirable.

## 1. Introduction

The extracellular acidification of the tumor’s microenvironment was reported to underlie not only proliferation and metastatization of cancer cells but also anticancer drugs resistance [1]. Indeed, the lactate production, following the increased anaerobic glycolysis, even under normal oxygen concentrations (Warburg effect), and the consequent pH decrease in the tumor microenvironment would impair the uptake of weakly basic cytotoxic drugs reducing their therapeutic effectiveness [1]. Furthermore, the activity of vacuolar H+-ATPases (V-ATPases), which is an ATP-dependent proton pump that generate the pH gradient across both plasma and intracellular organelles membranes, is enhanced in lysosomal type vesicles of cancer cells leading to both drugs sequestration into acidic vesicles and their extrusion [2]. Therefore, increasing the pH of the tumor microenvironment by targeting V-ATPases would represent an intriguing way to overcome the multi-drug resistance [3]. It has been demonstrated both in vitro and in vivo experiments that higher proton pump inhibitors (PPIs) doses than those used to block H+/K+-ATPase on gastric parietal cells can also inhibit V-ATPases, modulate tumor acidification and restore chemotherapeutic sensitivity in drug-resistant cancer cell [4,5,6]. 

Because acidic microenvironment is necessary for PPIs transformation into active molecules, PPIs should be delivered at intermittent high dose to achieve an anti-tumor effect. In vivo experiments showed that tumor pH shifts toward neutrality after esomeprazole treatment and returns to acidity within 48 h after treatment discontinuation [5]. Moreover, a dose of about 2.5 mg kg^−1^ in animal model, which is comparable to that used in humans for the Zollinger–Ellison syndrome (160–240 mg/day), was tested to revert tumor microenvironment acidity without evidence of systemic toxicity [1,4]. 

These preclinical experiments also confirmed the ability of high dose PPIs to counteract drug-resistance in human tumors and impair vitality of different tumor cells [5,6]. 

The first proof of concept clinical study investigated the chemosensitizer effect of PPIs in patients affected with osteosarcoma [7]. In this phase-2 trial, 98 patients received esomeprazole (60 mg/day) for two days before neoadjuvant treatment with methotrexate, cisplatin, and adriamycin. A higher percentage of tumor necrosis was detected in patients of the experimental compared to those of the control arm while the toxicity profile was similar between the two groups. Moreover, PPIs were tested in combination with docetaxel in advanced solid tumors with favorable results [8,9]. This way, to use existing drugs for new therapeutic applications, called “drug repositioning”, would represent a more tolerable and economically sustainable approach to cancer therapy [10,11]. 

Several cytotoxic agents can be redirected to an endothelial cell target by changing their dose and frequency of administration [12]. This ‘metronomic’ chemotherapy, which uses a frequent administration of low-doses without prolonged drug-free breaks, lately exhibited many additional mechanisms of action such as the stimulation of anti-tumor immunity as well as a direct inhibition of tumor cells growth [12]. As a consequence, many early clinical evaluations were carried out while several comparative studies are currently ongoing [13]. Particularly, metronomic capecitabine (mCAP) was tested as a single agent in patients with advanced gastrointestinal tumors with preliminary, encouraging results [14,15,16]. Although the best delivery schedule of mCAP has not yet been defined, mCAP at the dosage of 1500 mg/day, was recently demonstrated to be a moderately active, well-tolerated regimen as salvage chemotherapy in refractory, pretreated, metastatic gastrointestinal cancer [15,16]. Metronomic protocols were also showed to be suitable for in several combination therapies with targeted agents as well as maximum tolerated dose chemotherapy (chemo-switch) [17]. Moreover, as it can be easily combined with drug repositioning, metronomic chemotherapy is supposed to be an ideal companion to generate more potent, but less-toxic combination therapy for cancer patients [13]. Rabeprazole has a non-enzymatic metabolism, a low potential for drug interactions and a pharmacokinetics that is rather independent from inter-individual variations linked to polymorphisms of CYP2C19 and CYP3A4 [18]. 

According to all these findings the present phase II study was planned to evaluate both activity and safety of high dose rabeprazole combined with mCAP vs. mCAP alone, in patients with metastatic gastrointestinal (GI) cancer previously treated with several chemotherapy regimens. The possible interaction between the mCAP and rabeprazole was also investigated. 

## 2. Results

**Figure 1:**
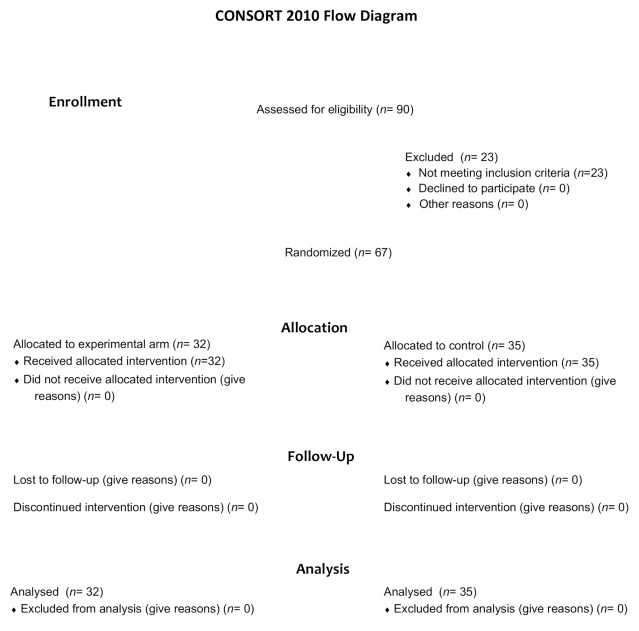
Consort diagram of the study depicting the process of patients’ randomization.

Between 10 February 2014, and 8 May 2019, a total of 90 patients were screened for eligibility (Figure 1). Sixty-seven patients underwent randomization, 32 were assigned to receive mCAP+rabeprazole and 35 to receive mCAP alone (intention-to-treat population). Each patient received the assigned study drugs according to the randomization schema and was evaluated for tumor response and toxicity. Baseline demographic and clinico-pathologic features were balanced between the two study groups (Table 1).

### 2.1. Efficacy

**Figure 2:**
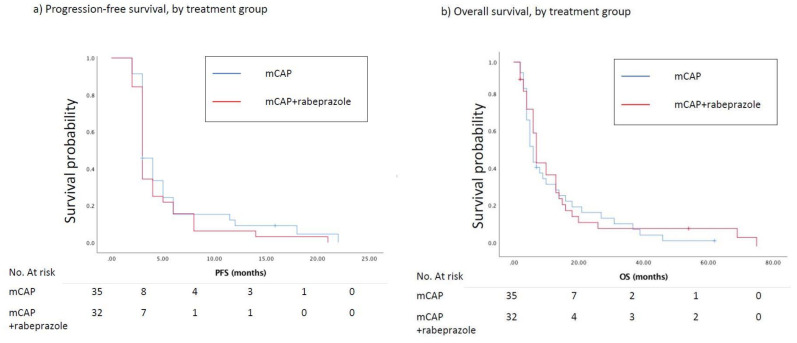
(**a**) Kaplan–Meier curves show progression-free survival (PFS) between patients treated with mCAP+rabeprazole (red line) and mCAP alone (blu line). The median PFS was 3.0 months in the mCAP+rabeprazole as well as in the mCAP alone group (HR = 1.22, 95%CI 0.75–2.00, *p* = 0.420); (**b**) Kaplan–Meier curves show overall survival (OS) between patients treated with mCAP+rabeprazole (red line) and mCAP alone (blue line). The median OS in the mCAP+rabeprazole was 7.0 (95%CI 3.9–10.1) months vs. 6.0 (95%CI 4.0–7.9) months in the mCAP alone (HR = 0.89, 95%CI 0.54–1.48, *p* = 0.664).

At the time of the analysis, the number of estimated events for PFS was 65. At the 3-months evaluation (primary end-point), 21 (66%) and 20 (57%) patients, respectively randomized to mCAP+rabeprazole and mCAP, experienced a progression of the disease (HR = 1.43, 95%CI 0.53–3.85; *p* = 0.477). The median PFS was 3.0 months in the mCAP+rabeprazole as well as in the mCAP alone group (HR = 1.22, 95%CI 0.75–2.00, *p* = 0.420) (Figure 2a). Nine (28%) and 11 (31%) patients reported a CB in the experimental and control arm respectively (RR = 0.85, 95%CI 0.29–2.44, *p* = 0.768). The median OS in the mCAP+rabeprazole was 7.0 (95%CI 3.9–10.1) months vs. 6.0 (95%CI 4.0–7.9) months in the mCAP alone (HR = 0.89, 95%CI 0.54–1.48, *p* = 0.664) (Figure 2b). A 3-year OS rate of 10% and 12% was reported in the mCAP-rabeprazole and mCAP group, respectively. Two patients are still being treated with palliative therapy. 

### 2.2. Safety

**Figure 3:**
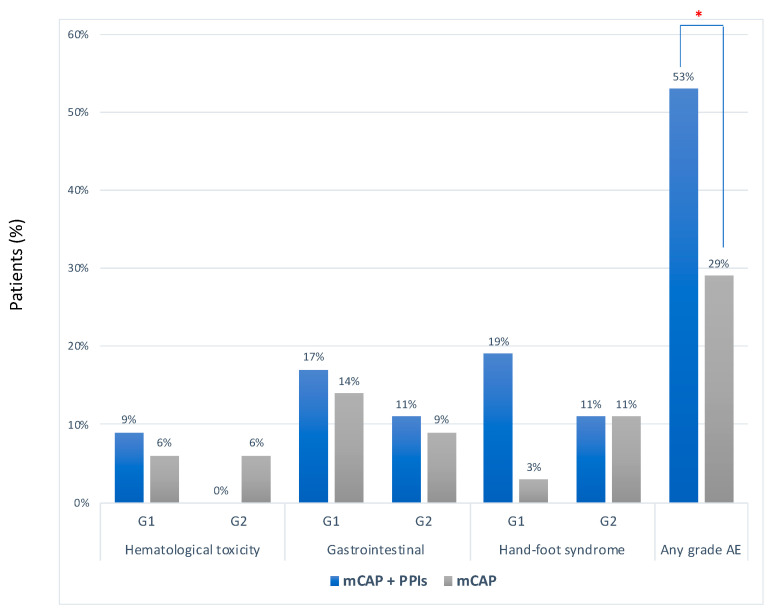
Frequency of adverse events according to CTCAE criteria between mCAP+PPIs (blue bar) and mCAP alone (grey bar). * *p* = 0.043.

The median number of cycles of chemotherapy was 2 (range 1–20) in the mCAP+rabeprazole group and 2 (2–22) in the mCAP group (*p* = 0.617) Overall, no severe adverse event was reported. However, adverse events of any grade occurred more frequently in the mCAP+rabeprazole than in the mCAP group (53% vs. 29%, OR 2.83, 95%CI 1.03–7.79; *p* = 0.043) (Figure 3). More detailed description of single adverse event was reported in Table S1.

### 2.3. Plasma Concentration of Capecitabine and Its Metabolites

Overall, the values of capecitabine, 5′-DFUR and 5-FU were below zero in the pre-dose analysis. There were no statistically significant differences between mCAP+rabeprazole vs. mCAP alone in the estimated plasma concentration of capecitabine and its metabolites after 4 and 8 weeks of treatment (Table 2). However, the Cmax value recorded for 5′-DFUR after 8 weeks was significantly increased when mCAP was combined with rabeprazole (mean ± SD, 1.26 ± 0.65 vs. 1.41 ± 1.25 µg/mL; *p* = 0.018).

## 3. Discussion

Interesting preclinical and clinical data pointed out the possibility to revert drug resistance of tumor cells by using high dose PPIs [4,5,6,7,8,9]. However, there were also recent data which reported negative retrospective results with the concurrent use of PPIs and capecitabine in different setting of patients affected with GI cancer [19,20,21]. Our study for the first time investigated in a randomized phase II trial the concurrent use of repurposed high-dose rabeprazole combined with low-dose metronomic capecitabine.

Metronomic capecitabine had previously been demonstrated as a moderately active, well-tolerated regimen in pretreated patients with metastatic GI cancer [14,15,16]. Moreover, preliminary data about 3 colorectal cancer patients included in our experimental protocol with mCAP and rabeprazole were remarkably positive [22].

Results of the present study showed a similar 3-months PFS rate in the two arms of treatment thus, the primary endpoint was not reached. However, the design of the study and the limited sample size could have prevented the detection of any significant difference between the treatment groups. However, some issues deserve a deeper consideration. First of all, survival curves of both treatment groups, are consistent with those reported from the literature in comparable patients affected with colorectal [23], gastric [24], and pancreatic cancer [25]. Indeed, a 3-year OS rate of 10% and 12% in the mCAP-rabeprazole and mCAP group respectively, was reported beyond expectations. In addition, although patients were frail and/or pretreated, the safety profile of metronomic schedule was good with no severe adverse events. Lastly, if our data cannot confirm the suggestive hypotheses of a positive interaction between high dose rabeprazole and metronomic capecitabine, nonetheless they can contribute to the recently opened debate about the concomitant use of repurposed PPIs and chemotherapy, particularly capecitabine. In fact, no detrimental effect was noticed by combining rabeprazole with capecitabine.

Conversely, before our study, negative results regarding the use of PPIs and capecitabine were reported [19,20,21]. In a retrospective post hoc analyses of the TRIO-013/LOGiC study, the concomitant use of PPIs with capecitabine and oxaliplatin (CapeOx) with or without lapatinib was evaluated in patients with ERBB2/HER2-positive metastatic gastroesophageal cancer [20]. Based on prescription refill data, PPI use was associated with a reduction of both median PFS (4.2 vs. 5.7 months, *p* < 0.001) and OS (9.2 vs. 11.3 months, *p* < 0.04) only in the CapeOx group. A comparable retrospective analysis was conducted in patients with early stage colorectal cancer treated with adjuvant capecitabine [21]. Five-year recurrence-free survival was 74% and 83% (HR = 1.89, *p* = 0.03) in patients with and without concurrent PPIs consumption respectively. These studies concluded that an increased gastric pH due to PPIs with a supposed consequent reduction of both dissolution and absorption of the capecitabine may explain the decrease in capecitabine activity.

Both these two studies share the limit of a retrospective evaluation in which confounding factors, such as the underlying condition to the PPIs prescription, could have acted. On the contrary, our study, where patients were randomly assigned to mCAP+rabeprazole or mCAP alone, revealed similar outcomes between the two arms but for the incidence of mild toxicity. Remarkably, a comparable estimated Cmax of capecitabine, 5′DFUR and 5-FU between the two treatment groups could be also observed. Therefore, the adjunct of rabeprazole, even delivered at high dosage did not seem to negatively influence the concentration of capecitabine and its metabolites. Actually, the Cmax recorded for 5′-DFUR after 8 weeks was even significantly increased by the association with rabeprazole, fact that might explain the slightly higher incidence of toxicity in this group. The lack of the urine metabolites testing as well as the short period of observation makes our pharmacokinetic analysis preliminary. Thus, both prospective clinical studies and confirmatory pharmacokinetic investigations are awaited. In detail, future clinical study should be addressed in these three following points (i) the choice of the PPI: A pre-clinical study has shown that lansoprazole exert the most significant anti-cancer effect both as a single agent [26] and in combination with chemotherapeutics [27,28], and therefore the future clinical study should be designed either using lansoprazole or lansoprazole compared to other PPI; (ii) the dose and the treatment schedule: both retrospective clinical studies and some case reports have shown that the dose may be lower than that used in our study (from 0.8 to 1 mg/kg) and taken each day [29,30,31]; (iii) a pre-clinical study has shown that PPI may allow to lower the dose of the chemotherapeutics [27] and therefore a further arm with a fixed dose of PPI and at least two different doses of capecitabine, or even other drugs, should be included. Of course, one arm treated with PPI alone, as it has been tested with important results in the treatment of metastatic breast cancer patients [9], should be included as well.

## 4. Material and Methods

Ethics Approval and Consent to Participate: The local independent ethics committee approved this study (Sapienza University, Department of Molecular and clinical medicine, Protocol number is 1169/2016 of 2014-02-19 EUDRACT 2013-001096-20). It conducted according to the principles of the Declaration of Helsinki and the International Conference on Harmonisation Good Clinical Practice guidelines. Trial registration, EudraCT, Number: 2013-001096-20, registered 9 February 2014. https://www.clinicaltrialsregister.eu/ctr-search/search?query=2013-001096-20. 

Consent for publication: a written informed consent was provided by each patient.

### 4.1. Patients

Patients with biopsy-proven/documented adenocarcinoma of the colon or rectum, stomach, or pancreas who had received one or more regimens of standard chemotherapy for metastatic disease and had a life expectancy >3months were eligible for randomization. In addition, patients had to be 18 years of age or older, to have adequate bone-marrow, liver, and renal function and to have an Eastern Cooperative Oncology Group (ECOG) performance status ≤2. Meanwhile, the presence of one of the following criteria excluded patients’ participation to the study protocol: GI tumors that can be treated with standard treatments, cardiovascular or CNS disease, previously untreated CNS metastases, pregnant or breast-feeding patients, organ dysfunctions that usually hinder the use of cytotoxic drugs, and/or substance abuse and any other psychological condition that may interfere with the evaluation of study results. All patients were randomized and treated at the Oncology Unit of Sant’Andrea University Hospital, Rome. Every cancer patient is commonly managed at our Institution by a multidisciplinary team, who provides for the starting and the following clinical evaluations. 

### 4.2. Study Design and Protocol

This is a randomized phase II study designed to detect the advantage of adding rabeprazole to mCAP as palliative treatment for patients with metastatic GI cancer, as previously described [32]. Eligible patients were randomly assigned 1:1 to capecitabine, 1500 mg/daily, continuously with or without rabeprazole 1.5 mg/kg bid, three days a week. The randomization was computer generated by a statistician not involved in data collection, who was called by phone at the time of assignment. The protocol was not interrupted until progression of disease, unacceptable toxicity or death occurrence. This study was designed with the primary endpoint of 3-months progression-free survival (PFS) rate. PFS was defined as the length of time from the first dose of study drug to the date of the first documented disease progression according to the Response Evaluation Criteria in Solid Tumors (RECIST), version 1.1, or death for any reason, with censoring at the date of the last contact for alive patients. Secondary endpoints included the evaluation of clinical benefit (CB), which reflects the proportion of patients with complete response (CR), partial response (PR) and stable disease (SD) and overall survival (OS). The crossover between treatment groups was not allowed before the final analysis of the primary end point. Safety was evaluated by considering overall incidence and severity of adverse events (AEs) as well as the seriousness and drug-relatedness of each event (CTCAE v.4.03). AEs with an onset during treatment, or within 28 days after the last dose of the study drug, were considered as having occurred during treatment. We grouped anemia, neutropenia, and thrombocytopenia in hematological toxicity; diarrhea, nausea, and vomiting in gastrointestinal toxicity. In the two protocol arms, plasma concentrations of capecitabine and its metabolites were determined at 2 h from the assumption of two tablet of capecitabine (1000 mg), corresponding to the tmax of the drug [33,34,35]. Plasma concentrations of capecitabine and its metabolites, 5′-deoxy-5′-fluorouridine (5′-DFUR) and 5- fluorouracil (5-FU), were evaluated in order to assess the potential interaction between capecitabine and rabeprazole. Blood samples (7 mL) were collected in vacutainers containing ethylenediaminetetraacetic acid (EDTA) as anticoagulant, then centrifuged and the plasma samples were stored at −20 °C until analysis. Plasma samples were collected at the start of the study, after 4 and 8 weeks of treatment. Plasma samples, calibration standard samples and quality-control (QC) samples were analyzed using a high-performance liquid chromatography (HPLC) tandem mass spectrometry (LC/MS-MS) analytical method. Plasma concentrations were reported in µg/mL (mean values and standard deviation (SD)).

### 4.3. Statistical Analysis, Sample Size

The study was designed to have 90% power to detect a hazard ratio for the 3-months PFS of 0.60 (a 40% reduction in risk) in the mCAP-rabeprazole group as compared with the mCAP group, with a one-sided type I error rate of 0.05. Given the treatment assignment ratio of 1:1, we calculated that 66 patients had to be enrolled in the study, and at least 60 events (progression disease) would have been required for the primary analysis. For PFS and OS, the stratified log-rank test was used independently at the 2-sided, 0.05 level of significance, and Kaplan–Meier estimates performed for the graphic representation of both findings. An exact logistic regression model was used to test for a difference in CB between treatments, with the model adjusted by the same factors used to stratify the primary analysis of PFS. Adverse events and laboratory abnormalities were summarized for all patients who received at least one dose of study drug. The χ^2^–test and t-test for unpaired data were applied to compare frequencies and means, respectively. SPSS statistical software, Version 24 (SPSS Inc. Chicago, IL, USA) was used.

## 5. Conclusions

The adjunct of high dose rabeprazole to mCAP in this study was not shown to increase the activity of mCAP alone in advanced GI patients. However, as PPI are being investigated worldwide as drugs to be repositioned in cancer treatment and also considering the limited sample size as well as the favorable safety profile of the combination in the present study, further clinical investigations are desirable.

## Figures and Tables

**Table 1:** Baseline characteristics of the intention-to-treat population.

Characteristics No. (%)		mCAP-Rabeprazole (*n* = 32)	mCAP (*n* = 35)	*p*
Age—years				
Median (range)		69 (39–85)	69 (38–87)	0.358
Sex				
	Male	22 (69)	20 (57)	0.449
	Female	10 (31)	15 (43)	
ECOG PS				
	0	14 (44)	9 (26)	0.197
	1	10 (31)	18 (51)	
	2	8 (25)	8 (23)	
Primary tumor site				
	Colon or rectum	28 (87)	28 (80)	0.134
	Stomach	0 (0)	4 (11)	
	Pancreas	4 (13)	3 (9)	
Site of metastasis				
	Liver	16 (50)	15 (43)	0.561
			
	Multiorgan	8 (25)	13 (37)	
	Peritoneum	8 (25)	7 (20)	
*n*. of prior regimens				
	1	6 (19)	5 (14)	0.349
	2	16 (50)	15 (43)	
	≥3	10 (31)	15 (43)	
Prior anticancer therapy				
	Fluoropyrimidine-based	14 (44)	16 (46)	0.244
	Anti-VEGF	16 (50)	10 (28)	
	Anti-EGFR	4 (12)	6 (17)	

**Table 2:** Estimates of plasma concentration (C) values recorded for capecitabine and its metabolites at tmax (2 h) with or without high dose rabeprazole.

Ctmax (μg/mL)	T1 (4 Weeks)			T2 (8 Weeks)		
	mCAP+rabe (22)	mCAP (20)	*p* Value	mCAP+rabe (22)	mCAP (20)	*p* Value
	(Mean ± Std.dev.)			(Mean ± Std.dev.)		
Capecitabine	0.58 ± 0.67	0.38 ± 0.46	0.315	0.38 ± 0.64	0.41 ± 0.32	0.160
5-DFUR	1.85 ± 1.52	1.43 ± 2.01	0.168	1.41 ± 1.25	1.26 ± 0.65	0.018
5-FU	0.29 ± 0.32	0.33 ± 0.62	0.270	0.31 ± 0.30	0.26 ± 0.34	0.968

Abbreviations: mCAP: metronomic capecitabine; rabe: rabeprazole; 5′-DFUR: 5‘-deoxy-5,-fluorouridine; 5-FU: 5-fluoruracil.

## Supplementary Materials

The following are available online at https://www.mdpi.com/2072-6694/12/11/3084/s1, Table S1: Adverse Events according to CTCAE criteria reported in the two groups.

## Abbreviations

Cmax	concentration at tmax
ECOG	Eastern Cooperative Oncology Group
GI	gastrointestinal
HR	hazard ratio
mCAP	metronomic capecitabine
OS	overall survival
PFS	progression free survival
PPIs	proton pump inhibitors
SD	standard deviation
RR	relative risk
