# Supplementary material for: Combination Therapy of High-Dose Rabeprazole Plus Metronomic Capecitabine in Advanced Gastro-Intestinal Cancer: A Randomized Phase II Trial
Source: Cancers (Basel). 2020 Oct 22;12(11):3084. doi: 10.3390/cancers12113084 (PMC7690608; doi:10.3390/cancers12113084)
Supplement: Supplementary file 1 [file cancers-12-03084-s001.pdf]

Supplementary Materials: Combination Therapy of High-Dose Rabeprazole Plus Metronomic Capecitabine in Advanced Gastro-Intestinal Cancer: A Randomized Phase II Trial

Michela Roberto, Adriana Romiti, Federica Mazzuca, Annalisa Milano, Chiara D'Antonio, Luana Lionetto, Rosa Falcone, Lidia Strigari, Maurizio Simmaco, Stefano Fais and Paolo Marchetti

Table S1. Adverse Events according to CTCAE criteria reported in the two groups.

Group	*n.* (%)	Anemia	Neutropenia	Thrombocytopenia	Diarrhea	Nausea	Vomiting
mCAP+rabeprazole	G1	2	1	0	4	2	1
	G2	0	0	0	3	1	0
mCAP	G1	2	0	0	1	1	1
	G2	1	0	1	2	1	0

Publisher's Note: MDPI stays neutral with regard to jurisdictional claims in published maps and institutional affiliations.

© 2020 by the authors. Licensee MDPI, Basel, Switzerland. This article is an open access article distributed under the terms and conditions of the Creative Commons Attribution (CC BY) license (http://creativecommons.org/licenses/by/4.0/).
